# Methodological Concerns Regarding a Recent Meta‐Analysis of Direct Oral Anticoagulants Versus Vitamin K Antagonists in Non‐Valvular Atrial Fibrillation

**DOI:** 10.1002/clc.70341

**Published:** 2026-05-04

**Authors:** Omar Khalid Samir, Nikita Datta

**Affiliations:** ^1^ Faculty of Medicine Port Said University Port Said Egypt; ^2^ Dhaka Medical College Dhaka Bangladesh

Dear Editor,

We read with great interest the systematic review and meta‐analysis by Nasir et al. assessing the comparative efficacy and safety of direct oral anticoagulants (DOACs) versus vitamin K antagonists (VKAs) in patients with non‐valvular atrial fibrillation (NVAF) [1]. The authors combined data from 11 studies with more than 814 000 patients and reported reductions in cerebrovascular ischemic events and major bleeding with DOACs. We would, however, like to offer a few comments that may help better interpret these findings.

First, and most critically, there is a possible discrepancy concerning the included study “Costa [24]”. In Table 1 of the meta‐analysis, this study is described as a US retrospective cohort of 83 182 NVAF patients with diabetes newly started on rivaroxaban or warfarin between 2010 and 2019. However, upon checking the cited reference, we found that it is a study of rivaroxaban versus warfarin in a matched sample of 13 510 obese patients with acute venous thromboembolism, which is an entirely different population and a significantly smaller sample size [2]. Given that this study contributed ~10% of the total patient population and was also the only study excluded in the sensitivity analysis that changed the all‐cause mortality result from no significant difference (RR: 1.02, 95% CI: 0.34–3.13, *p* = 0.97), to a significant risk reduction in the DOAC group (RR: 0.79, 95% CI: 0.63–0.98, *p* = 0.03), this discrepancy impacts the integrity of the pooled estimates.

Additionally, the study “Clara [20]” in Table 1 appears to carry a mismatched reference. The authors describe a Spanish retrospective cohort of 41 560 AF patients receiving DOACs versus acenocoumarol between November 2011 and December 2015, which matches a published study by Rodríguez‐Bernal et al. [3]. However, the cited reference “[20]” corresponds to a different paper, which is a meta‐analysis by Bonanad et al. comparing DOACs versus warfarin in octogenarians [4].

Second, pooling all four DOACs, which are apixaban, rivaroxaban, dabigatran, and edoxaban, into a single treatment arm may obscure clinically meaningful intra‐class differences. Many studies suggest that these drugs differ in their bleeding profiles, with apixaban having a more favorable safety profile compared to rivaroxaban [5, 6, 7, 8]. Therefore, subgroup analyses by individual DOAC, especially for the safety and bleeding outcomes, would enhance the clinical applicability of the findings.

Third, the search strategy was limited to PubMed, ClinicalTrials. gov, and the Cochrane Library. Although these are key resources, the authors did not search large databases, including EMBASE, Scopus, and Web of Science (WoS). Evidence has shown that MEDLINE/PubMed alone identifies only 86.6% of relevant articles, and that a PubMed‐EMBASE combined search increases coverage to ~90%, with some of the included studies being only retrievable in EMBASE. This can potentially lead to bias in the pooled estimate due to missed studies [9].

Fourth, the analysis pooled data from randomized controlled trials (RCTs) and retrospective observational cohorts together to calculate risk ratios. Mixing study designs without can introduce confounding, as observational data are more prone to bias. Stratifying by study design would make this conclusion robust.

Fifth, extreme heterogeneity (*I*² = 100%) in all‐cause mortality requires more than leave‐one‐out analysis of a miscited study. Meta‐regression using Table 2 covariates, such as CHA₂DS₂‐VASc, follow‐up, and diabetes, should have been attempted to explain these variances more clearly [10].

Lastly, we noted a mismatch between figure captions and content. For example, Figure 3 is captioned for major bleeding risk, yet the results section attributes this figure to cerebrovascular ischemic events.

The findings of this meta‐analysis are clinically meaningful. However, the concerns mentioned above suggest the results should be interpreted with caution. Addressing these points in future research will strengthen the evidence for anticoagulant selection in NVAF.

## Conflicts of Interest

The authors declare no conflicts of interest.

## Data Availability

Data sharing not applicable to this article as no datasets were generated or analysed during the current study.
